# Psychosine enhances the shedding of membrane microvesicles: Implications in demyelination in Krabbe’s disease

**DOI:** 10.1371/journal.pone.0178103

**Published:** 2017-05-22

**Authors:** Ludovic D’Auria, Cory Reiter, Emma Ward, Ana Lis Moyano, Michael S. Marshall, Duc Nguyen, Giuseppe Scesa, Zane Hauck, Richard van Breemen, Maria I. Givogri, Ernesto R. Bongarzone

**Affiliations:** 1 Department of Anatomy and Cell Biology, College of Medicine, University of Illinois, Chicago, Illinois, United States of America; 2 Department of Medicinal Chemistry and Pharmacognosy, College of Pharmacy, University of Illinois, Chicago, Illinois, United States of America; 3 Departamento de Química Biologica, Facultad de Farmacia y Bioquímica, Universidad de Buenos Aires, Buenos Aires, Argentina; Cornell University, UNITED STATES

## Abstract

In prior studies, our laboratory showed that psychosine accumulates and disrupts lipid rafts in brain membranes of Krabbe’s disease. A model of lipid raft disruption helped explaining psychosine’s effects on several signaling pathways important for oligodendrocyte survival and differentiation but provided more limited insight in how this sphingolipid caused demyelination. Here, we have studied how this cationic inverted coned lipid affects the fluidity, stability and structure of myelin and plasma membranes. Using a combination of cutting-edge imaging techniques in non-myelinating (red blood cell), and myelinating (oligodendrocyte) cell models, we show that psychosine is sufficient to disrupt sphingomyelin-enriched domains, increases the rigidity of localized areas in the plasma membrane, and promotes the shedding of membranous microvesicles. The same physicochemical and structural changes were measured in myelin membranes purified from the mutant mouse Twitcher, a model for Krabbe’s disease. Areas of higher rigidity were measured in Twitcher myelin and correlated with higher levels of psychosine and of myelin microvesiculation. These results expand our previous analyses and support, for the first time a pathogenic mechanism where psychosine’s toxicity in Krabbe disease involves deregulation of cell signaling not only by disruption of membrane rafts, but also by direct local destabilization and fragmentation of the membrane through microvesiculation. This model of membrane disruption may be fundamental to introduce focal weak points in the myelin sheath, and consequent diffuse demyelination in this leukodystrophy, with possible commonality to other demyelinating disorders.

## Introduction

Krabbe’s disease is a genetic leukodystrophy where mutations in the galactosyl-ceramidase gene cause the aberrant accumulation of undigested galactolipids [1]. One of these, galactosyl-sphingosine, also known as psychosine, has been notoriously indicated as the main sphingolipid underpinning demyelination by the killing of oligodendrocytes (reviewed in [2]). Although prior studies from our laboratory showed that psychosine accumulates in lipid rafts, and is sufficient to introduce fundamental changes in their architecture and behavior [3], a direct role–if any- of psychosine in the damage to myelin sheaths in Krabbe’s disease has not been fully addressed.

For effective demyelination, psychosine would not only need to accumulate in the myelin domain but it also would have to significantly disrupt intra- and intermembrane interactions that keep myelin lamellae compacted. Cis and trans interactions between several glycosphingolipids (GSL) and myelin proteins are fundamental for the production of the stable and mature myelin compacted sheath [4]. Because of their chemical structure, composed of a hydrophobic sphingoid base and a hydrophilic inverted cone polar head group, GSL show high melting points that favor lipid compaction into lipid rafts. In addition, the size of the polar head groups (sphingomyelin < galactosyl-ceramide < psychosine << sulfatides <<< gangliosides) introduces space restrictions that greatly impact on the lateral mobility, fluidity and curvature of biological membranes [5–8]. Through these mechanisms, abnormal levels of GSL may facilitate the shedding and destruction of biological membranes, including myelin.

Being an inverted cone cationic sphingolipid, psychosine has the ideal chemistry to introduce significant changes in membrane behavior not only by affecting lipid rafts [3], but also by altering membrane fluidity [9]. Hence, we speculated that in addition to disrupting lipid rafts, the progressive accumulation of psychosine in myelin restricts lateral mobility in the myelin membrane, reduces membrane fluidity, and increases the chances of myelin destruction by membrane shedding. Here, we show that aberrant levels of psychosine affected lipid organization of sphingomyelin-enriched submicrometric domains [10], increased membrane rigidity, and facilitated the microvesiculation and shedding of myelin, providing a deeper insight into the mechanism of demyelination of Krabbe’s disease.

## Materials & methods

### Ethics statement

The use of twitcher mice and the experimental studies were approved by the Animal Care and Use Committee of the University of Illinois at Chicago (Protocol Number 15–101). Breeder Twitcher heterozygous mice (C57BL/6J, twi/+) were originally purchased from Jackson Laboratory, maintained and treated under standard housing conditions with animal care and use committee protocols of our institution. Male and female Twitcher mice were used indistinctly. Mice were anesthetized by isofluorane, and killed by cervical dislocation.

### Oligodendroglial cultures

The primary oligodendrocyte cultures were performed as described previously [11]. Briefly, mice pups were sacrificed at P2-P4, cortex was collected and dissociated and glial cells were cultured in FBS-based media for 14–15 days before enrichment of oligodendrocytes by shaking method. The cells were then grown in oligodendrocyte-specific media (OL media) for 4–6 days and these media were collected to isolate microparticles for flow cytometry. CG-4 cells (a kind gift from Dr Jean de Vellis, UCLA) were plated at 3,500 cells/cm^2^ in 10cm^2^ Corning® Costar® plates and cultured in CG-4 media as described previously [12]. After 4 days of culture, medium was replaced either by fresh CG-4 medium (proliferating cells) or by oligodendrocyte medium (differentiating medium as detailed in [11]). Cells were cultured for 3 additional days prior to lipid treatment and analysis by flow cytometry. Enriched cortical oligodendrocytes and CG-4 cells were washed with PBS and incubated overnight at 37°C with CO_2_ 5% with indicated concentrations of psychosine or the corresponding concentration of vehicle (DMSO) in CG-4 or oligodendrocyte media.

### Erythrocyte isolation, labeling and imaging

Blood was collected on the day of the experiment either from heart before sacrifice and placed into EDTA-coated Eppendorf or through the retro-orbital plexus by Heparin-coated capillaries (Fischer Scientific). The blood was diluted in Hank’s Buffer Salt Solution (HBSS; pH 7.4) and sample was centrifuged at 133 *g* for 2 min to separate erythrocytes (red blood cells, RBCs) from plasma. Pelleted RBCs were washed and centrifuged twice before resuspension in 10 volumes HBSS. For immobilization, coverslips were coated with 0.1 mg/ml 70–150 kDa poly-lys incubated for 7 min then diluted 1:1 with HBSS for 7 min before aspiration. After drying, coated-coverslips were used within 4h. RBCs were immobilized onto coated coverslips at 20°C for 4 min, after which suspension was removed and replaced by fresh medium to allow further RBC spreading for another 4 min before lipid labeling. Wild-type RBCs were pre-incubated with various concentrations of psychosine or corresponding amounts of vehicle (DMSO) at 37°C for 30 min in the presence of an equimolar ratio of defatted Bovine-serum albumin (DF-BSA; Sigma), washed by centrifugation at 133 *g* for 2 min and re-suspended into fresh medium before immobilization onto coated coverslips. Data were reported after normalization to vehicle (DMSO). RBCs were labeled with BODIPY-lipids (dipyrrometheneboron difluoride ring, Thermo Fischer Scientific) after immobilization on coated-coverslips. Briefly, cells were rinsed in HBSS and incubated at 20°C for 15 min with either 0.75 μM of BODIPY-SM, 1 μM of -PC, or 1 μM -GM1 in HBSS containing equimolar DF-BSA, and finally washed with HBSS [13]. RBCs labeled for cholesterol analysis by FRAP utilized a modified protocol from Leppimaki et al, [14]. Briefly, TopFluor-cholesterol (TpF-chol, 23-(dipyrrometheneboron difluoride)-24-norcholesterol, Avanti Polar Lipids) was complexed to methyl-β-cyclodextrin at a molar ratio of defatted-BSA (DF-BSA):sterol:cyclodextrin, 1:1:8, in HBSS. This solution was sonicated in a water bath for 3 X 10 min, vortexed 2 min in-between each sonication, and finally centrifuged at 15,000 rpm for 30 min at 20^°^C. The complex was then quantified to assess recovery of lipid probe using a spectrofluorometric plate reader (excitation 485 nm, emission 535nm). RBCs preincubated with 2μM psychosine or DMSO vehicle, immobilized on coverslips, were incubated with TpF-chol-cyclodextrin complex at a calculated concentration of 25 μg/ml sterol for 3 min at 20^°^C. For confocal imaging, the coverslip was placed bottom-up into a Lab-Tek chamber (Nunc^TM^) and examined in the green channel with a Zeiss LSM 510 or a LSM 710BIG confocal microscope using a plan-achromat 63x NA 1.4 oil immersion objective. For time-lapse experiments, RBCs were immobilized on coated-coverslips, labeled with BODIPY-SM and imaged with psychosine added on the stage. Fluorescence measurement of BODIPY-SM inserted in RBCs was determined on cells lysed with Triton X-100 (0.1%) and detected by a spectrofluorometer DTX-880 (Beckman-Coulter, Pasadena, CA) at 485nm-excitation and 535 nm-emission wavelengths. For Laurdan imaging, RBCs were incubated with 1.5 μM Laurdan at 37°C for 30 min, centrifuged (130 *g* for 5 min) and immobilized onto coated coverslips before image acquisition.

### Psychosine extraction and analysis by mass spectrometry

Samples were extracted by methanol with acetic acid 0.5% (v/v) upon shaking for 1h at room temperature, centrifugation at 15,000 g for 10 min, and the supernatant was collected and analyzed using liquid chromatography tandem mass spectrometry as described previously [15]. Briefly, positive ion electrospray tandem mass spectrometry with selected reaction monitoring was performed using a Shimadzu LCMS-8050 triple quadrupole mass spectrometer equipped with a Shimadzu Nexera UHPLC system.

### Fluorescence recovery after photobleaching (FRAP)

RBCs were examined under confocal microscope as described above. A field with several RBCs upon low laser illumination was selected and a 5-μm^2^ square regions of interest (ROI) was defined. The ROI was placed in the middle of adjacent RBCs in order to cover 2.5 μm^2^ per erythrocyte (<5% cell surface). After recording the initial intensity (fluorescence before bleaching; I_i_), the ROI was briefly photobleached (90–100% of laser power) to achieved a minimal residual fluorescence intensity (I_bleach_; ~10–30% of I_i_). Fluorescence recovery time (I_t_) was recorded for 30 sec after returning to a low laser illumination. ROI that moved during this interval were excluded (<20%). Values were calculated as (I_t_-I_bleach_)/(I_i_-I_bleach_) and fitted to a single exponential to extrapolate mobile fraction at infinite time recovery (in %).

### Osmotic resistance and hemolysis assays

RBCs were incubated in HBSS of different osmotic concentrations (from an isotonic medium of 320 mOsm down to pure H_2_O of 0 mOsm) for 30 min at 37°C before analysis of hemolysis. Hemolysis was evaluated by hemoglobin release into supernatant after centrifugation at 133 *g* for 2 min. Complete hemolysis was induced with 0.2%Triton X-100 and used as control [16, 17].

### Myelin extraction

Brains were homogenized in 0.3 M sucrose 20 mM Tris-HCl buffer (pH 7.5), 1 mM EDTA, 1 mM DTT, 100 μM PMSF using a pestle homogenizer for ~ 10 strokes. Samples, solutions and centrifugations were all maintained on ice. Myelin was purified as described elsewhere [18]. Myelin was resuspended in a small volume of water, aliquoted and centrifuged at 12,000 *g* for 15 min for storage as a pellet to be kept at 4°C for 2 days or at -20°C for longer periods of time. Myelin was incubated with psychosine at indicated concentrations at 37°C for 90 min, centrifuged at 12,000 *g*, and the supernatant was collected for microvesicle analysis. For Laurdan imaging, myelin was resuspended with 1.5 μM of Laurdan at 37°C for 60 min, centrifuged at 12,000 *g* for 15 min and resuspended on coated coverslips before image acquisition by multiphoton microscopy.

### Flow cytometry

Ten μL of blood were stained on ice for 30 min in 100 μL Flow Buffer solution (Ringer solution with 0.2% BSA and 2 mM EDTA) containing 1 μg/ml final concentration of APC-conjugated anti-CD235a to recognize RBCs and 1 μM final concentration CellTrace^TM^ CFSE proliferation kit to probe vesicles and exclude cell debris (ThermoFischer Scientific). Wild-type blood incubated with 5 μM calcium ionophore A23187 (Sigma) and 5 mM CaCl_2_ (Merck) was used as positive control to induce vesiculation [19] (data not shown). For enriched cortical oligodendrocytes, CG-4 cells and myelin, supernatant was collected, centrifuged at 100 *g* for 10 min at room temperature to pellet cells and debris bigger than 5 μm. The supernatant was centrifuged at 21,000 *g* for 20 min at 4°C to pellet the microparticles and stained for 1h on ice in 100 μl Flow Buffer solution containing 1 μM final concentration CellTrace^TM^ CFSE and 1:60 dilution of Annexin V Pacific Blue conjugate (ThermoFischer Scientific). This solution was centrifuged at 21,000 *g* for 20 min at 4°C, washed with flow buffer solution, spun and resuspended in 100 μl of flow buffer solution. Stained microparticles were diluted in Buffer solution to reach 0.5 ml containing 10,000 fluorescent beads (CountBright^TM^, Molecular Probes) to standardize the quantification of RBCs and particles (called altogether events). The events were detected by a Gallios Cytometer analyzer (Beckman-Coulter) equipped with lasers at 488 nm and 638 nm wavelengths. 1,000 fluorescent beads were counted per sample. Events were identified by size through comparison with beads from the Flow cytometry size calibration kit (Molecular Probes). Data were analyzed by Kaluza software (Beckman-Coulter) and reported as percentage of control (untreated cells or vehicle).

### Anisotropy measurement

Anisotropy was measured by fluorescence polarization of 1-(4-(Trimethylammoniumphenyl)-6-Phenyl)-1,3,5-Hexatriene (TMA-DPH; Sigma) [20]. RBCs (100x10^6^ cells) or myelin (100–200 μg protein) were suspended in Phosphate Buffered Saline (PBS) solution and incubated with 1 μM TMA-DPH for 10 min and fluorescence was acquired with a PTI Quantamaster spectrofluorimeter and excitation and emission wavelengths were, respectively, 355 nm and at 430 nm. The steady-state fluorescence polarization was expressed as the anisotropy (*r*) according to the following equation previously described [21]: *r* = (I_vv_-GI_vh_)/(I_vv_+2GI_vh_), where I_vv_ and I_vh_ correspond to the simultaneous fluorescence acquisition with both parallel (I_vv_) or perpendicular (I_vh_) positions of the excitation and emission polarizers, respectively. The G (= I_hv_/I_hh_) stands for the instrumental correction factor. Each point of anisotropy corresponds to an average of 3 measures with 10 iterations each.

### Lipid packing analysis by Laurdan multiphoton microscopy

Images were acquired with Ultima Prairie two-photon microscope (Coherent laser, Prairie technologie) at 800 nm for excitation and emission collected simultaneously using two detectors with filters at 440 nm (ordered membrane) and 500 nm (disordered membrane). The calculation of GP was performed with a custom-written macro for ImageJ adapted from [22]. Briefly, GP is determined by the equation: GP = (I_ordered_-G*I_disorded_)/(I_ordered_+G*I_disorded_), where I = intensity signal; G = correcting factor. GP image was displayed in pseudocolors (rainbow RGB; blue-red lookup table) with the threshold pixels colored in black (background) and the color range set from GP -1 (blue, disordered) to +1 (red, ordered). Pseudocolored pixels were merged with the mean intensity image using hue-brightness-saturation (HSB) color space. GP was assigned to hue (color), mean intensity to brightness and saturation set to 1. The result producing an image with (dis)ordered intensities as measured by the lookup scale. A grayscale intensity image was acquired for quantification. The histogram of intensity in ROI at the center of the RBC in a grayscale image was analyzed by ImageJ and plotted into Gaussian distribution, normalized to the total number of pixels. A mean of the GP values was generated by non-linear fitting curve of Gaussian distribution and the % of control was plotted for comparison between treatments. Cell surface (cm^2^) was measured from pixels of Laurdan images and converted by ImageJ software corresponding to PLK-free surface of immobilized RBCs.

### Thin layer chromatography

RBCs were treated with 0–20 μM of psychosine for 30 min at 39°C. After washing and lysis in H_2_O, lipids were extracted by chloroform:methanol (2:1) and separated by thin layer chromatography (TLC) in chloroform:methanol:CaCl_2_ 15 mM (65:35:8; v:v)[23] and revealed by charring densitometry (180°C for 5 min) after staining with 10% cupric sulfate in 8% ortho-phosphoric acid [24]. Quantification was performed by densitometry.

### Electron microscopy

Tissue samples were collected and processed for transmission electron microscopy as described [25].

### Statistical analysis

Values are mean ± SEM of at least 3 independent experiments. Statistical analysis was measured using GraphPad (San Diego). Data were analyzed by two-tailed unpaired *t* test (confidence interval 95%) or by ANOVA test followed by Bonferroni multiple-comparison test to analyze for all possible comparisons, where applicable. NS, not significant (>0.05); *, *p* <0.05; **, *p* <0.01; ***, *p* <0.001.

## Results

### Membrane vesiculation of myelin in Krabbe’s disease, a stereotypic genetic sphingolipidosis

**Fig 1:**
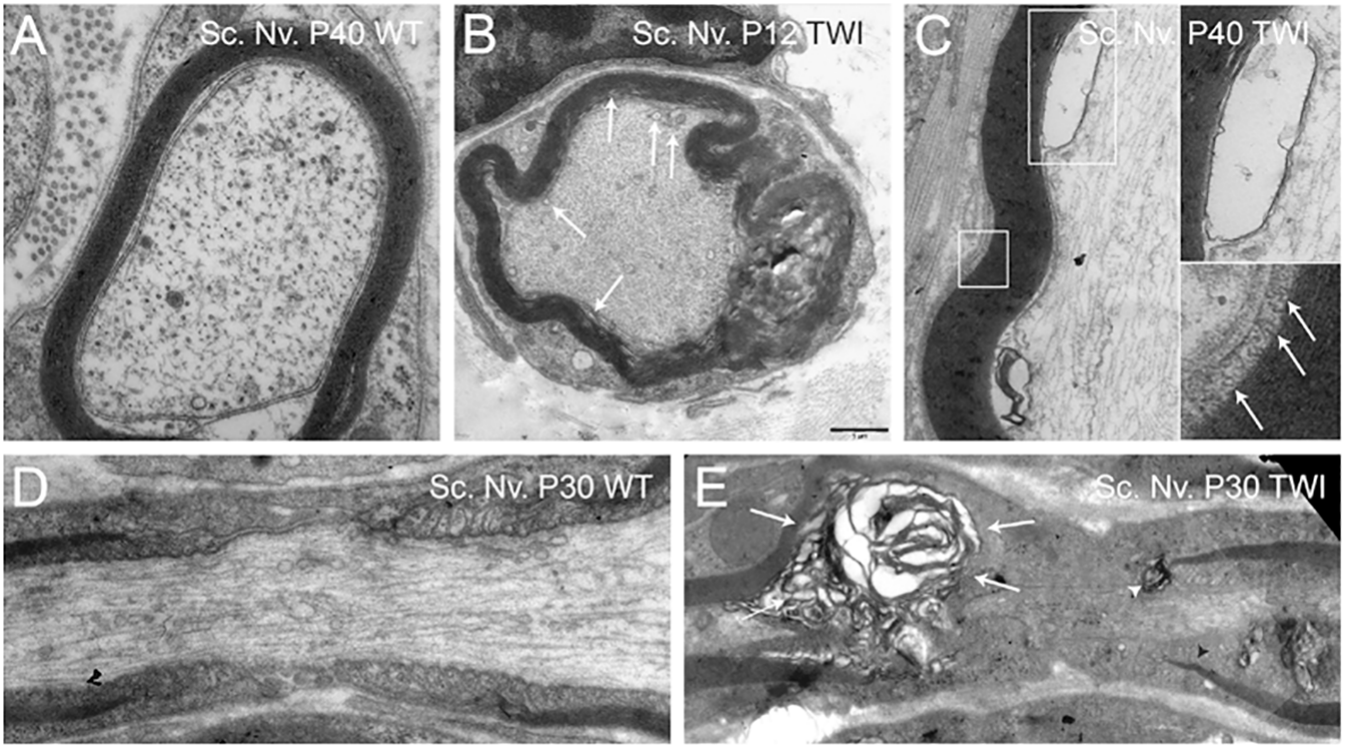
Microvesiculation of myelin in the Twitcher mouse. Electron microscopy micrographs of myelinated axons from peripheral nervous system shows myelin damage and vesicle association (arrows) demonstrated at sciatic nerves (Sc. Nv.) in presymptomatic P12 Twitcher (**B**) and late stage P40 Twitcher (**C,** top inset: large vesicle expanded. Bottom inset: myelin protruding from lamella) as compared to healthy wild-type (**A**). The ultrastructure of the Node of Ranvier in sciatic nerve of a healthy wild-type (**D**) *vs* Twitcher (**E**) presenting vesicular disruption of myelin lamellae (**E,** arrows) and region specific paranodal normalcy with myelin rupture at one side of the paranode region (white arrowhead) whereas other regions of the paranode were intact (dark arrowhead). D, E are composite images from multiple photographs. Bar = 1 μm, applies to all panels.

Krabbe's disease is a sphingolipidosis caused by the deficiency of galactosylceramidase (GALC) activity. GALC is a lysosomal hydrolase that normally degrades galactosyl-ceramides and galactosyl-sphingosine (psychosine); its deficiency causes the toxic accumulation of undegraded substrates [1]. Psychosine, which is considered to cause the death of oligodendrocytes and demyelination in Krabbe patients, is a sphingolipid that accumulates in lipid rafts originating from the brain, where it modulates associated signaling and endocytosis [3, 26]. To test our hypothesis that local GSL accumulation increases membrane rigidity and induces membrane shedding, we used the Twitcher mouse. This mouse model carries a mutation in codon 339 of the *GALC* gene, leading to GALC deficiency, psychosine accumulation, and recapitulation of neurological signs and histopathology observed in human Krabbe patients [27, 28]. Examination of peripheral nerves prepared from postnatal (P) days 12, 30 and 40 Twitcher mice revealed not only the traditional profiles of diffuse demyelination (Fig 1B and 1E), but also the presence of abundant vesicular material at the apposition of the myelin and axoplasma membranes (Fig 1B arrows, 1C, boxes) as well as at the outer tongue of myelin membranes (Fig 1C, arrows in boxed image). Altered profiles of myelin compatible with vesiculation can also be found in several electron microscopy studies of sphingolipidoses such as metachromatic leukodystrophy [29–31], Krabbe's disease [32], Tay-Sachs' disease [33], Farber's disease [34], and in multiple sclerosis [35], underlining an underestimated involvement of membrane alterations including shedding in several demyelinating conditions.

### Psychosine reduces specific lipid planar mobility, increases focal rigidity, and induces plasma membrane shedding

**Fig 2:**
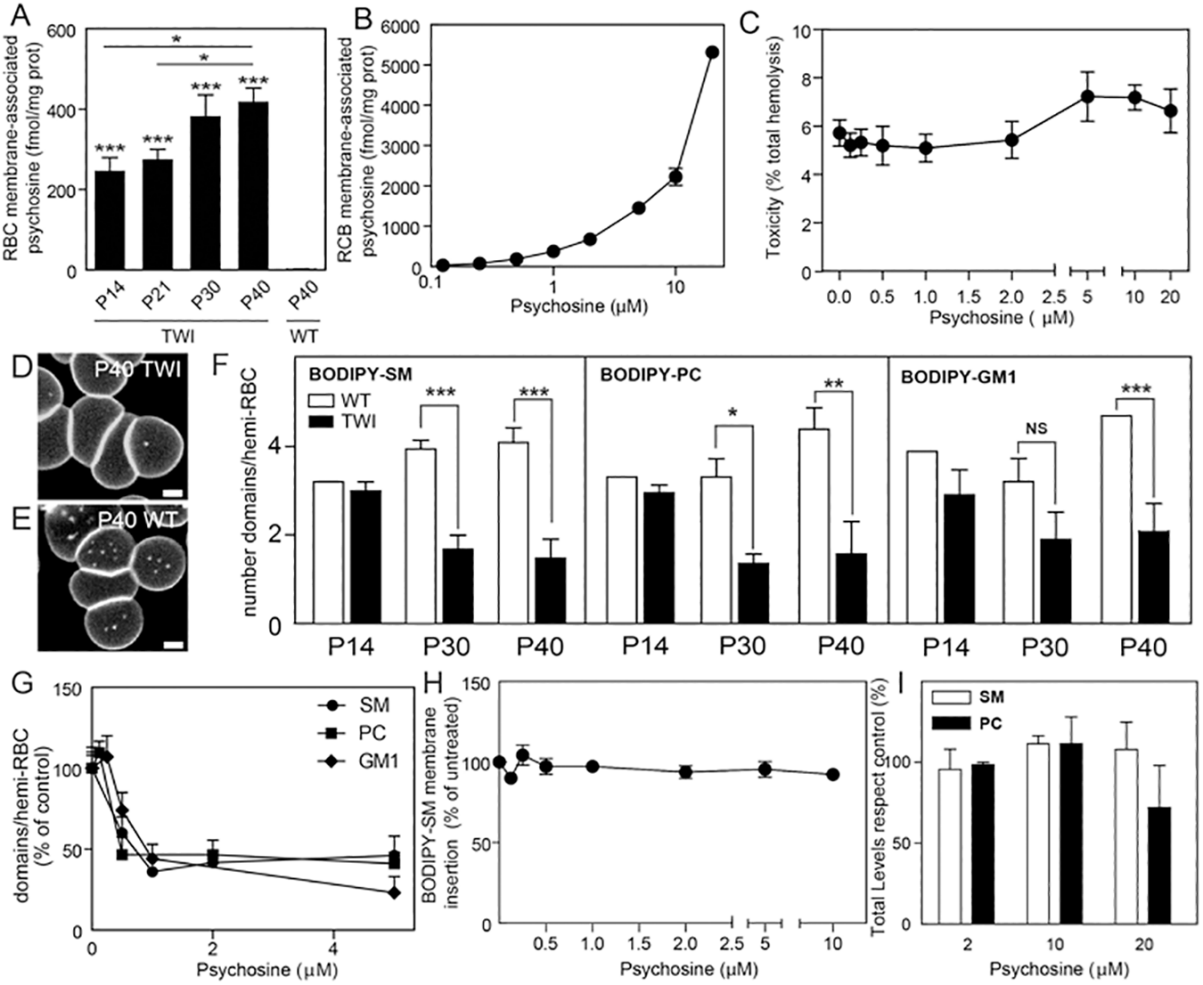
Psychosine destabilizes submicrometric lipid domains in red blood cells. **A)** Red blood cells (RBCs) from Twitcher mice and healthy littermates collected at different days of age (P14-P40) were analyzed for psychosine content. Psychosine in Twitcher RBCs was significantly higher at all ages as compared to healthy control, with psychosine levels increasing with age (n = 3 in 4 independent experiments). **B)** Wild-type RBCs incubated with 0–10 μM of psychosine, washed and processed for psychosine extraction and quantification. Data from 6 independent experiments. **C)** Hemolysis was measured after incubation with 0–20 μM psychosine. **D, E)** Confocal images of P40 RBCs labeled with BODIPY-SM showed a decrease of BODIPY-SM domains in Twitcher (**D**) as compared to wild-type (**E**). Scale bars, 2 μm. **F)** Isolated-RBCs collected from P14-P40 were dyed with BODIPY-SM, -PC and -GM1 and labeled sub-micrometric domains were counted by confocal microscopy. There was no difference in domains of Twitcher *vs* wild-type RBCs from mice below 30 days of age, while domains were significantly decreased in Twitcher RBCs at P30 and P40 (N = 109–924 in 3–6 independent experiments). **G)** Domains were counted in P40 wild-type hemi-RBCs after exposure to 0–5 μM of psychosine and labeled with BODIPY-SM, -PC or -GM1. **H)** RBCs were incubated with various concentrations of psychosine before labeling with BODIPY-SM and membrane fluorescence determined. **I)** RBCs were incubated with various concentration of psychosine. Sphingomyelin (SM) and phosphatidylcholine (PC) were extracted and separated on thin layer chromatography and quantified by densitometry. All aforementioned psychosine incubations were 30 minutes at 37^°^C. The results are mean ± SEM. NS, not significant (>0.05); *, *p* <0.05; **, *p* <0.01; ***, *p* <0.001.

First, we determined psychosine's perturbation effects using purified preparations of RBCs as a model to study lipid dynamics in plasma membranes [10]. Psychosine's effects were measured in RBCs prepared from affected Twitcher and healthy cells exposed to exogenous psychosine. In both conditions, psychosine was found enriched in purified preparations of RBC plasma membranes (Fig 2A and 2B), correlating with disease progression [36, 37], and without significant damage (i.e. hemolysis) to RBCs (Fig 2C). Submicrometric domains, ranging between 0.2–2 μm were identified by high-resolution confocal microscopy of fluorescent analogues of GSL (BODIPY-labeled GSL), as previously validated in human RBCs [10]. BODIPY-labeled psychosine probes are not available but we used BODIPY-labeled sphingomyelin (SM), phosphatidylcholine (PC), and ganglioside GM1 probes for qualitative and quantitative analyses of plasma membrane submicrometric domains [38]. The abundance of BODIPY-SM, BODIPY-PC and BODIPY-GM1 labeled domains significantly decreased in Twitcher cells with disease progression, particularly in animals at the terminal stage of the disease (Fig 2D–2F). Lipid incorporation experiments further demonstrated that psychosine was sufficient to induce similar reductions in the plasma membrane domains of healthy erythrocytes (Fig 2G). Disruption of sub-micrometric domains was neither caused by deficient insertion of BODIPY-lipid probes in the presence of psychosine (Fig 2H), nor by reduction of endogenous lipid levels as quantified by thin layer chromatography (Fig 2I).

**Fig 3:**
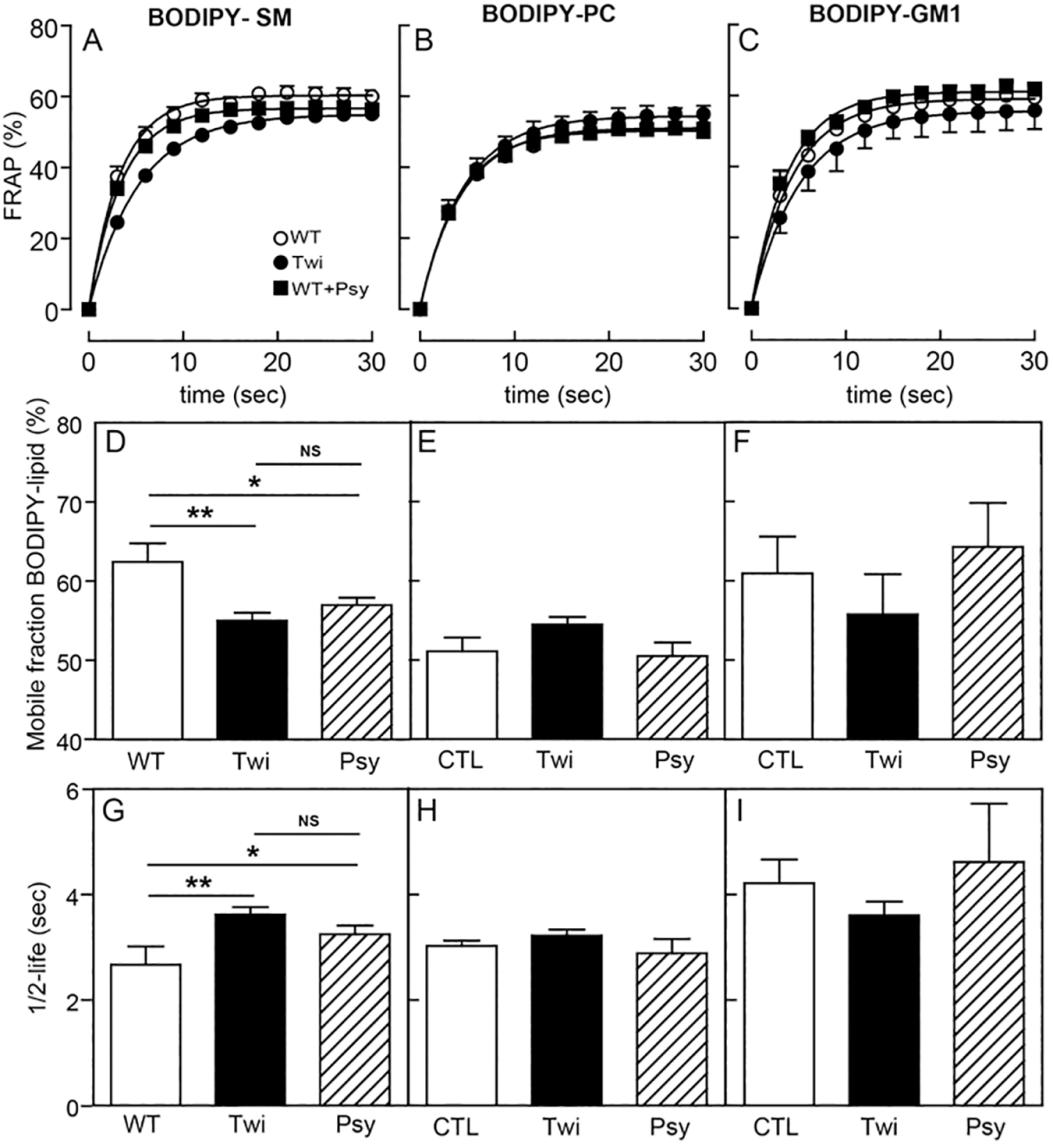
Psychosine reduces sphingomyelin lateral mobility. **A-C)** Delayed time to recover from photobleaching by BODIPY-SM (**A**), BODIPY-PC (**B**) and BODIPY-GM1 (**C**) (N = 61–648 in 1–4 independent experiments). **D-F)** The mobile fraction of BODIPY-SM (**D**) but not that of BODIPY-PC (**E**) and BODIPY-GM1 (**F**) in Twitcher and wild-type RBCs treated with psychosine were significantly lower than wild-type RBCs. **G-I)** Similarly, the half-life of FRAP signal was increased for BODIPY-SM (**G**) but not for BODIPY-PC (**H**) and BODIPY-GM1 (**I**) in Twitcher and wild-type RBCs treated with psychosine, evidencing an effect of psychosine in the plasma membrane and an interaction with sphingomyelin. All aforementioned psychosine incubations were 30 minutes at 37^°^C Results are mean ± SEM. NS, not significant (>0.05); *, *p* <0.05; **, *p* <0.01; ***, *p* <0.001.

Plasma membrane lipids are distributed between those clustering into immobile submicrometric domains and the mobile fraction, including those moving individually or in clusters within the membrane plane [13]. The latter can be indirectly studied by fluorescence recovery after photobleaching (FRAP), which is primarily used to study lateral diffusion of plasma membrane constituents [39]. This measure is used to functionally reflect lipid compartmentalization by observing a restriction of lateral diffusion after the photobleaching of a field at a specific size. Indeed, a free, unrestricted and homogenously organized molecule should display 100% recovery by FRAP measurement. However, when a pool of the plasma membrane molecules is constrained, it shows decreased lateral diffusion and will have restricted mobility. We and others have previously reported that lipids were partially restricted in their mobility as an indirect evidence of plasma membrane compartmentalization in different eukaryotic cell lines and human RBCs [38, 40–42]. Accordingly, we confirmed here that this restriction of mobility exists in murine RBCs, as the mobile fraction in healthy RBC was ~65% for the three BODIPY-lipids. FRAP analyses showed a significant (~15%, p value < 0.05) decrease of mobile BODIPY-SM in the plasma membrane of Twitcher RBCs (Fig 3A and 3D), demonstrated by a significant increase of the half-life mobility (Fig 3G). Treatment of healthy RBCs with exogenous psychosine also reduced the mobility of BODIPY-SM (Fig 3A and 3D). Similar FRAP analyses for BODIPY-PC and BODIPY-GM1 showed non-significant changes in their mobility in Twitcher RBCs or after incubation with psychosine (Fig 3).

The observed effect of psychosine on BODIPY-SM mobile fractions and half-life suggest that lipid rafts may additionally be altered. SM and cholesterol are the most abundant lipid species found in lipid rafts, and previous work has shown psychosine to correlate with cholesterol in lipid raft fractions [3]. As such, we measured cholesterol dynamics in RBCs pre-incubated with psychosine using FRAP analysis. TopFluor-cholesterol complexed with cyclodextrin evidenced a significant decrease in mobile fraction, with increased half-life mobility in psychosine treated RBCs (S1 Fig). This result suggests a transient interaction of psychosine with cholesterol, which could lead to more restricted mobility and downstream consequences in membrane organization and cellular signaling. Altogether, these results show that psychosine destabilizes not only the number and organization of sub-micrometric domains in the plasma membrane, but also reduces the planar mobility of specific lipids such as SM and cholesterol (Table 1).

**Table 1:** Psychosine reduces sphingomyelin and cholesterol lateral mobility.

Probes	Mobile Fraction (%)			Half-life (sec)		
	WT	Twi	Psy	WT	Twi	Psy
**BODIPY-SM**	62.4 ± 2.3	55.0 ± 1.0**	57.0 ± 0.9*	2.7 ± 0.3	3.6 ± 0.1**	3.2 ± 0.2*
**BODIPY-PC**	51.1 ± 1.7	54.4 ± 0.9	50.5 ± 1.7	3.0 ± 0.1	3.2 ± 0.1	2.9 ± 0.3
**BODIPY-GM1**	60.9 ± 1.3	55.8 ± 2.2	64.3 ± 2.7	4.2 ± 0.4	3.6 ± 0.2	4.6 ± 1.1
**TpF-CHO**	41.5 ± 2.3	n.d.	36.1 ± 1.3**	3.6 ± 0.4	n.d.	8.7 ± 0.6**

Twitcher (Twi) and wild-type (WT) RBC (preincubated with DMSO or 2 μM psychosine) were immobilized and incubated with the indicated BODIPY or TpF-fluorescent lipid probe for FRAP measurement. Mobile fraction and half-life were derived from the fluorescence intensity at ‘infinite’ time after photobleaching, calculated by monoexponential fitting.

Values are mean ± SEM (n = 61–648, 2–4 experiments)

*p<0.05

**p<0.01 as compared to WT.

n.d., non-determined.

**Fig 4:**
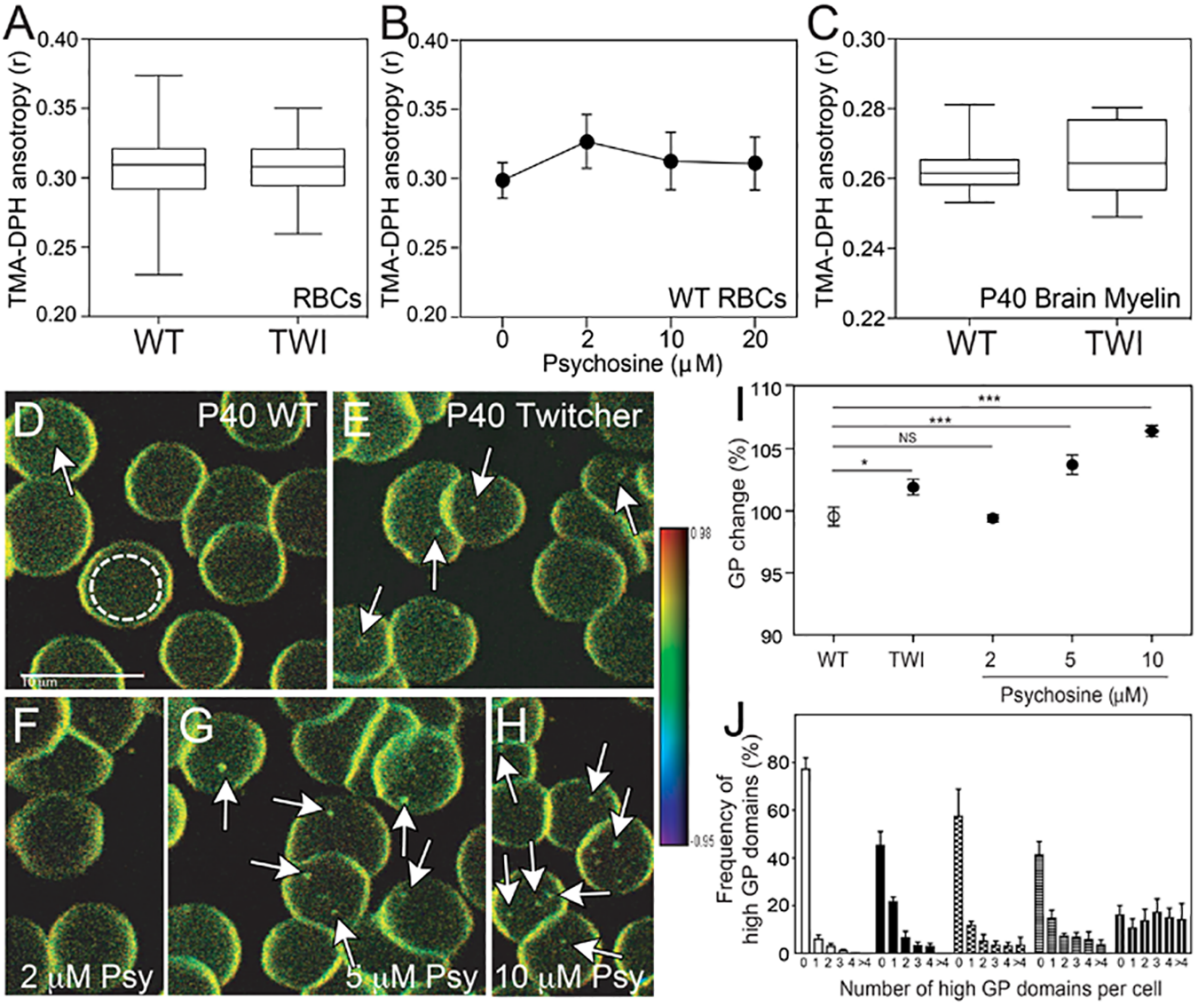
Psychosine increases rigidity in focalized areas of the RBC plasma membrane. **A-C)** Overall fluidity in RBCs (**A,B**) and purified P40 brain myelin (**C**) measured by TMA-DPH is not affected by psychosine or in Twitcher mice. Wild-type, Twitcher, and wild-type RBCs preincubated with increasing concentrations of psychosine were analyzed by TMA-DPH anisotropy. **D-H)** Multiphoton excitation microscopy imaging of Laurdan in P40 wild-type (**D**), Twitcher (**E**), and wild-type RBCs treated with 2 (**F**), 5 (**G**) or 10 μM of psychosine for 30 minutes at 37^°^C. (**H**). GP images are in pseudo-colors with the range indicated by the color bar going from fluid (blue/green) to rigid (yellow/red). Scale bar, 10 μm. **I)** GP value from the center of RBCs were analyzed as represented inside the white dashed circles in (**D**) and plotted as % of change from wild-type values. **J**) Distribution of domains of high GP and high-rigidity. Results are mean ± SEM of samples N = 68–257 in 3–4 independent experiments. NS, not significant (>0.05); *, *p* <0.05; **, *p* <0.01; ***, *p* <0.001.

The reduction of SM and cholesterol lateral diffusion and disruption of sub-micrometric domains predicted that psychosine would induce changes in the overall fluidity of the plasma membrane, which is largely defined by the dynamics and organization of its constitutive lipids [43]. Alternatively, changes in fluidity may be restricted to focal areas in the membrane. To study these two possibilities, we used anisotropic spectroscopy of trimethyl-ammonium diphenylhexatriene (TMA-DPH) to infer changes in overall membrane fluidity [44] and generalized polarization (GP) of Laurdan for direct focal fluidity measurements [22]. TMA-DPH anisotropy in Twitcher and psychosine-treated wild-type erythrocytes, as well as treated wild-type and Twitcher myelin showed an absence of significant changes in overall membrane fluidity (Fig 4A–4C). Furthermore, there was no overall osmotic-dependent membrane fragility observed in Twitcher erythrocytes (Data non-shown). In contrast, psychosine induced focal increases of ordering/rigidity in Twitcher erythrocytes and wild-type cells exposed to the lipid (Fig 4D–4H, arrows), with significantly increased GP values as measured by Laurdan fluorescence (Fig 4I). Furthermore, the frequency of high GP domains per cell was significantly increased in Twitcher cells as well as in control cells after psychosine treatment (Fig 4J). The discrepancies observed between these distinct measurements of membrane fluidity are explained by the unique detection thresholds of TMA-DPH and Laurdan to membrane changes. The bulk measurement of RBC membranes by TMA-DPH likely diluted the detection of localized membrane changes due to anisotropy distortion not measured in the central region of the cell. These same changes were detectable by Laurdan due to its deeper insertion into cell membranes, including centralized regions, as well as its homogenous insertion with emission-shift measurements remaining independent lipid phase state. These results demonstrate that psychosine effects on the plasma membrane are not generalized to the entire membrane, but rather are restricted to local areas of accumulation, in agreement with our previous findings [3]. Psychosine effects included the induction of domains of high rigidity, which could potentially force other sub-micrometric domains (i.e. those enriched with BODIPY-lipids) to disassemble (Fig 2F).

**Fig 5:**
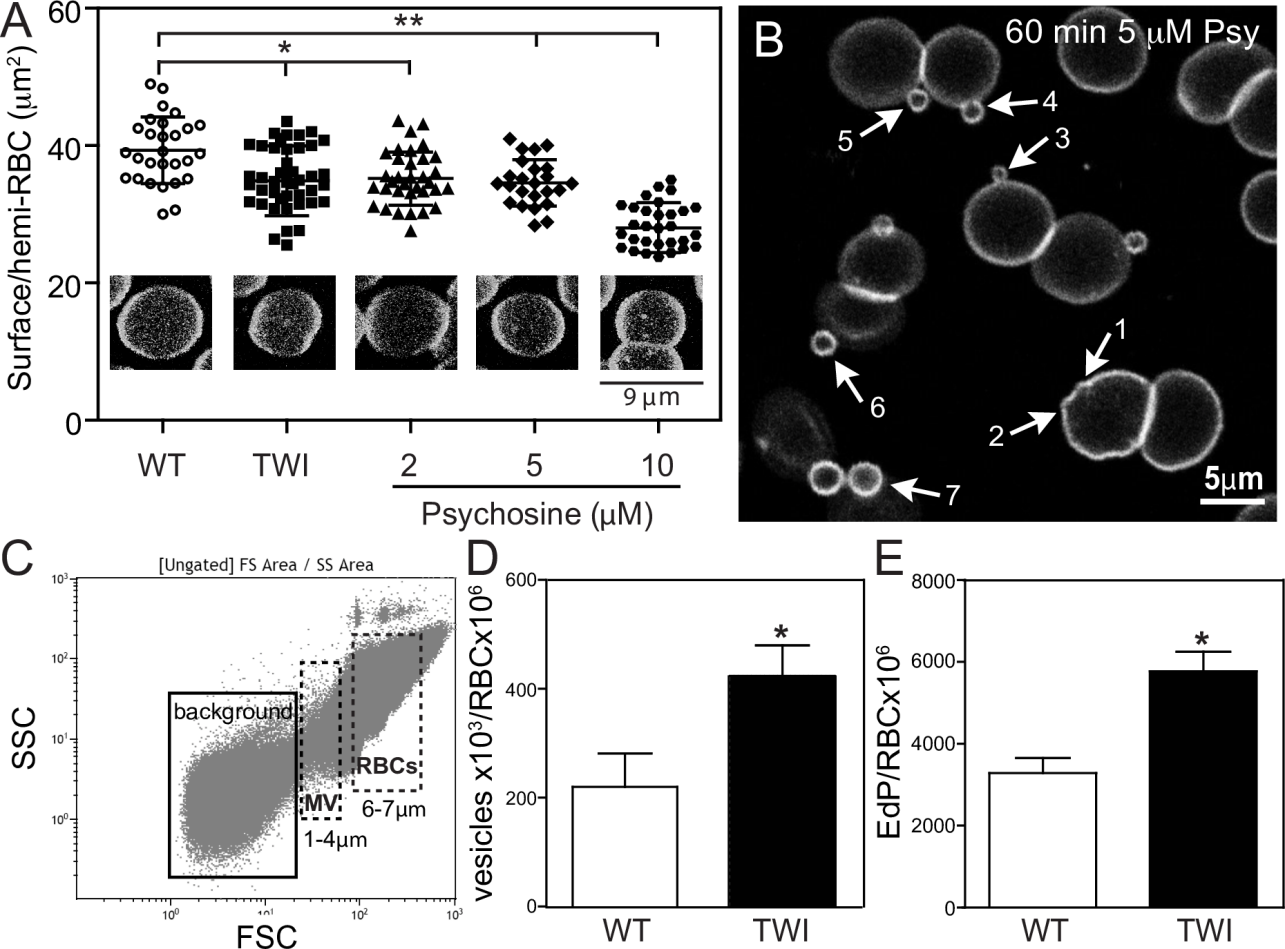
Psychosine affects cell surface area by vesiculation. **A)** RBCs from P40 TWI or WT pre-incubated with psychosine for 30 minutes at 37^°^C were labeled with BODIPY-SM, plated on poly-lysine coated slides and their surface area was measured by confocal microscopy. Twitcher cell surface area was significantly decreased, an effect also elicited in wild-type cells after exposure to 2–10 μM of psychosine (N = 28–44 in 3 independent experiments). **B)** RBCs labeled with BODIPY-SM were exposed to psychosine during a 60 min on stage incubation. Vesicles appeared at the surface following different stages of budding, from membrane outward bending (#1, #2), to small (1–2 μm, #3, #4) and large (2–4 μm #5 to #7) vesicle budding. **C-E)** Flow cytometry graphs plotting side (SSC) and forward scatter (FSC) of events with a gate selected for size 1–4 μm (microvesicles, mv) and 6–7 μm (RBCs) (C) showed a significant increase in Twitcher with respect to wild-type cells (D). Analysis of RBC CD235a confirmed the erythrocyte origin of the vesicles (expressed as erythrocyte-derived particles, EdP in E). Results in D and E were normalized by total RBCs counted in the 6–7 μm region from 3 independent experiments. Results are mean ± SEM. NS, not significant (>0.05); *, *p* <0.05; **, *p* <0.01; ***, *p* <0.001.

The increase of rigid areas in the membrane plane in response to focal increments of psychosine underlines the possibility that abnormal local membrane tensions at the edge of submicrometric domains could lead to membrane shedding [45]. To study this, an analysis of cell area was first performed on immobilized erythrocytes via confocal microscopy, confirming a significant decrease in the average cell surface area of Twitcher cells in comparison to control cells (36.1 ± 5.1 *vs* 40.6 ± 4.8 μm^2^, respectively) (Fig 5A). Psychosine was found to reduce the cell surface area to a similar degree (Fig 5A). Shrinkage of erythrocytes has been observed in various disease conditions [17, 19, 46] and attributed to increased membrane curvature and shedding (i.e. vesiculation). These particles range in size from several nanometers up to ~4 μm and may have roles in the propagation of pathogenic cues [47]. Confocal microscopy imaging on wild-type RBCs labeled with BODIPY-SM, revealed abundant areas of increased curvature after on-stage incubation with psychosine (Fig 5B, arrows 1 and 2), leading into the budding and shedding of 0.5–4 μm diameter vesicles (Fig 5B, arrows 3–7, and S1 Movie). Control RBCs exposed to vehicle (DMSO) showed minimal protrusion (S2 Movie).

To quantify this process, we used laser-assisted flow cytometry to count the number of vesicles released from Twitcher and control cells (Fig 5C) [19, 46, 48]. Due to the complex composition of blood, the threshold for detection of smaller microvesicles over background noise was optimized to include those from 1–4 μm. We determined that membrane shedding in the selected vesicular gate (1–4 μm) was doubled in Twitcher cells (Fig 5D). Double labeling of vesicles for the vesicular marker CFSE and the erythrocyte marker CD235a confirmed a two-fold increase of erythrocyte-derived particles (EdP) in Twitcher cells (Fig 5E). Altogether, these results show that psychosine introduces focal regions of rigidity in the RBC plasma membrane, affecting its curvature and facilitating membrane shedding.

### Psychosine increases membrane rigidity and shedding of oligodendroglial cells and myelin

**Fig 6:**
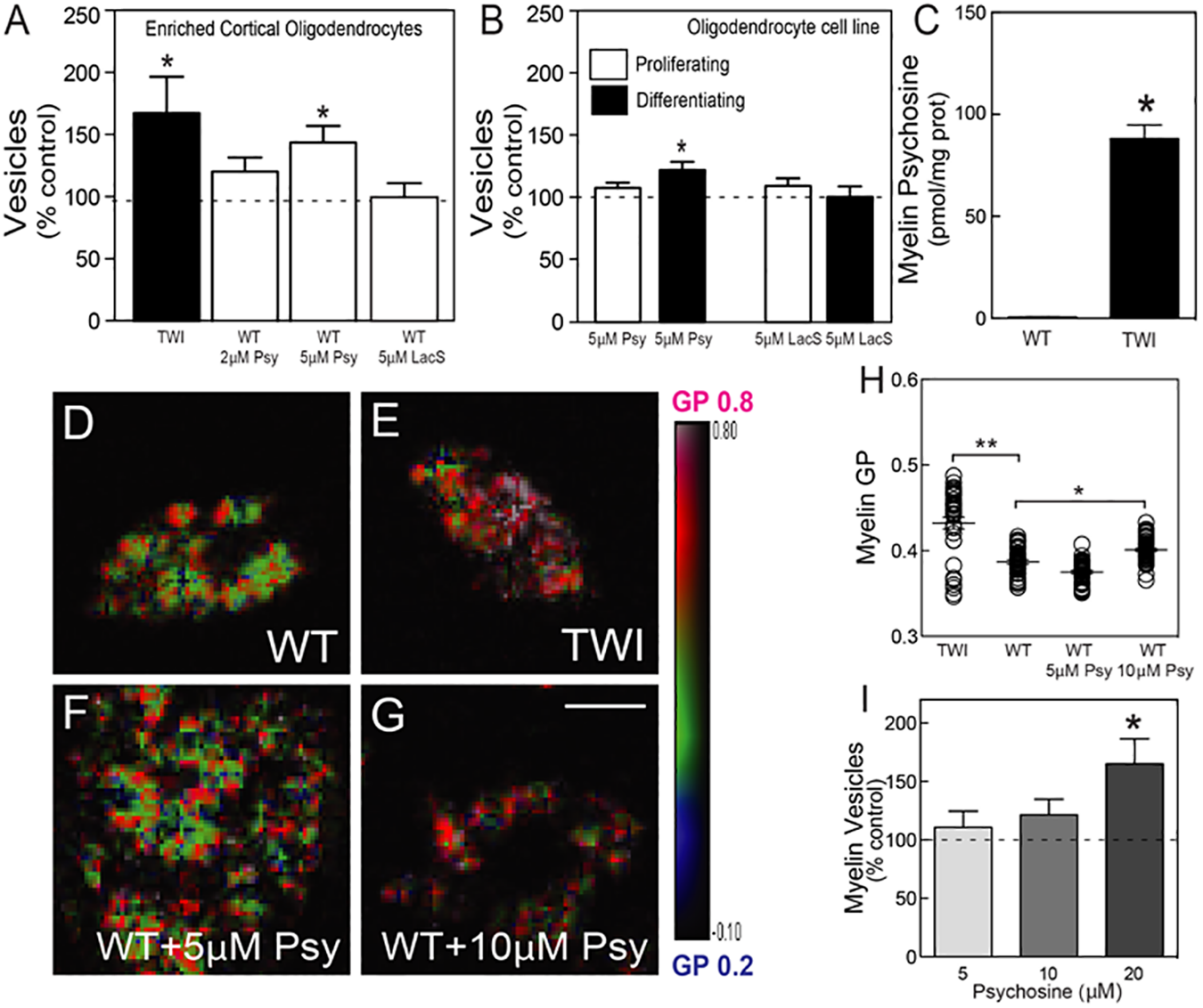
Membrane rigidification and vesiculation of Twitcher oligodendrocytes and myelin. **A, B)** Flow cytometry quantification of vesicles (0.5–3 μm) from enriched cortical oligodendrocytes from Twitcher or wild-type mice incubated with 2–5 μM of psychosine overnight at 37^°^C (**A**) and from CG-4 cell cultures in proliferating or differentiating conditions (**B**). Incubation with lactosyl-sphingosine (LacS) did not significantly promote the release of vesicles in brain-purified oligodendrocytes (**A**) and CG-4 cells (**B**). **C-H)** Psychosine content in myelin extracted from P40 wild-type and Twitcher mice (**C**). Brain myelin extracted from P40 wild-type (**D**), Twitcher (**E**) and wild-type treated with 5 (**F**) or 10 μM of psychosine for 90 minutes at 37^°^C (**G**) were incubated with Laurdan and analyzed by multiphoton microscopy (**H**). **I)** Vesicles were analyzed by flow cytometry from purified wild-type myelin after incubation with 5–20 μM of psychosine for 90 minutes at 37^°^C. Results are expressed as percentage from control levels (i.e. myelin incubated with vehicle) of 3–7 independent experiments and presented as the mean ± SEM. NS, not significant (>0.05); *, *p* <0.05; **, *p* <0.01; ***, *p* <0.001.

A prediction derived from our results is that Twitcher oligodendrocytes, which, along with myelin, constitute the primary pathological target in Krabbe’s disease, will be prone to similar psychosine-induced membrane shedding. We tested this idea in cultures of cortical Twitcher oligodendrocytes, acutely prepared from newborn brains and differentiated for one week before analysis of microvesicle release. The isolation of vesicles from this cell population allowed for a more sensitive detection of smaller vesicles (0.5–3 μm). Flow cytometry analysis revealed a ~170% increase of the gated 0.5–3 μm microvesicles released in the supernatant by Twitcher oligodendrocytes in respect to control levels (Fig 6A). Furthermore, a dose-dependent effect of psychosine was measured indicating that 5 μM psychosine was sufficient to induce the release of microvesicles from wild-type oligodendrocytes at levels similar to Twitcher cells (Fig 6A).

In Krabbe’s disease, most of white matter pathology occurs once oligodendrocytes are differentiated and producing myelin [49, 50]. Myelinating oligodendrocytes are generated by differentiation of highly proliferative progenitors, but little is known about deficits in GALC mutant progenitors in this disease. To study if cell cycling status renders oligodendroglial cells more or less sensitive to the shedding effects of psychosine, a bipotential oligodendrocyte-type 2-astrocyte progenitor cell line (CG-4 cell line) [51] was used to generate proliferative (i.e. under the pressure of platelet derived growth factor) or differentiating (i.e. under the pressure of thyroid hormone and absence of mitogens) oligodendroglial cells, subsequently exposed to psychosine. Psychosine induced significant levels of microvesicle release in differentiated but not proliferative oligodendrocytes (Fig 6B), suggesting that the membrane of proliferating progenitors is less vulnerable to psychosine. Experiments using a control lipid (lactosyl-sphingosine, LacS) showed lack of significant shedding (Fig 6B).

Demyelination in Krabbe’s disease has been correlated for a long time with the progressive increase of psychosine in the affected brain [43, 52]. However, the mechanism involved in the process of demyelination is unclear. Based on the previous experiments, we predicted that local increases of psychosine in the mutant myelin would lead to increase of myelin rigidity, and microvesiculation. In fact, psychosine is ~100-fold higher in myelin purified from P40 Twitcher brains in respect to levels found in age-matched control myelin (Fig 6C). Local fluidity measurements (changes in GP Laurdan emission), but not overall fluidity measures (TMA-DPH anisotropy, Fig 4B), clearly showed that Twitcher myelin is significantly more rigid than control myelin (Fig 6D, 6E and 6H). Similar increases of local membrane rigidity were measured after incubating control myelin with increasing concentrations of psychosine (Fig 6F, 6G and 6H). Furthermore, incubation with psychosine induced the progressive release of microvesicles from control myelin membrane preparations (Fig 6I).

Altogether, our results support a pathogenic model where the local concentration of psychosine modifies myelin fluidity towards a more rigid status and leads to fragmentation via shedding of microvesicles. This mechanism may be a key contributor to demyelination in Krabbe’s disease.

## Discussion

Our study advances new evidence for a toxicity mechanism by which the accumulation of the lysosphingolipid psychosine damages myelin by rigidification of its membranes, providing for the first time a mechanistic insight in how psychosine directly contributes to the process of demyelination of Krabbe’s disease. By using three different experimental systems including RBCs (non-myelinating, organelle free cells), oligodendrocytes, and myelin, our results support a model where the insertion of psychosine molecules in concentrations that represent those found in peripheral blood and nervous tissue from sick Twitcher mice, results in architectural and dynamic disorganization and destabilization of the membrane. By studying membrane disorganization in the RBC system, we found that psychosine elicited lower lateral mobility of sphingomyelin and cholesterol, which correlated to areas in the plasma membrane with higher focal rigidity, and lead to membrane shedding. This process did not appear restricted to a specific cell type, but was rather dependent on psychosine itself, as these effects were also observed in oligodendrocytes isolated from the mouse cortex, and the CG-4 oligodendroglial cell line in conditions of high psychosine content.

As postulated by the lipid raft theory, GSL are essential contributors to lateral membrane heterogeneity and are important for membrane bioactivity and cellular functions [6]. It is reasonable to hypothesize that the insertion of aberrant levels of psychosine in cellular membranes initiates cellular toxicity by affecting membrane-based mechanisms. In our previous studies, we found that psychosine is enriched in and disrupts the architecture of lipid rafts [3], underlining the impact of this lipid on membrane structure. Additionally, a recent study showed that an enantiomer of psychosine elicited similar levels of toxicity to the natural form of psychosine, underlining that psychosine’s toxicity involves at least lipid-lipid interactions [53]. Support for this notion was attained from membrane swelling of protein-free liposomes after insertion of the enantiomer [53]. Our present study adds to these reports by supporting a model where psychosine is sufficient to disrupt lipid-lipid homeostasis in cell membranes, affecting selected lipids. For example, the association of GSL with cholesterol is the foundational idea of the lipid raft theory [54], and clearly one that is altered in Krabbe’s disease [3]. Indeed, the presence of psychosine decreased cholesterol extractability, indicating that the two lipids interact within the membrane [53], contributing to decreased membrane fluidity [55]. Central to the proposed mechanism of psychosine-mediated loss of myelin structure is the confirmation of significant levels (~100-fold with respect to control levels) of psychosine in myelin in the brain of affected Twitcher mice.

Changes in membrane fluidity have been also measured in other sphingolipidoses such as Niemann-Pick [56–58], GM1 gangliosidosis [59], in chemical (LPC) induced demyelination [60] and a recent work using rotating-polarization coherent anti-stokes raman scattering showed disordered myelin in sciatic nerves from sick Twitcher mice [61]. Such changes contribute to increased cellular vulnerability in sphingolipidoses [43, 62] but may also bear importance in myelin vulnerability in other conditions such as Multiple Sclerosis [63] and Alzheimer’s disease [64, 65]. In this aspect, our study provides with the first evidence that psychosine not only accumulates in myelin membranes but is sufficient to cause myelin loss by microvesiculation.

Although our results support a model where lipid-lipid homeostasis is targeted by psychosine, it does not rule out other potential direct or indirect interactions between psychosine and integral proteins, peripheral proteins, and/or the submembranous cytoskeleton, which in conjunction with the psychosine-lipid effects described above would enhance toxicity *in vivo* [9]. Future analyses will interrogate the role of direct and/or indirect psychosine-protein interactions in demyelination.

Fluidity, which directly impacts membrane bending, is a key biophysical property that underpins the adaptation of structure to function. Because each lipid has a specific shape dictated by its chemical structure, the accumulation of specific lipids is sufficient to induce changes in membrane curvature [66]. In this respect, our results show that psychosine increases focal rigidity in myelin, which in the context of disease, may introduce focal areas where the myelin sheath weakens and facilitates its disintegration during demyelination. *In vivo* electron imagery has clearly shown increased vesiculation in myelinated axons of affected patients and animal models [29–35]. Such changes in membrane curvature may also help explain other membrane alterations found in Krabbe’s disease such as axonal and dendritic membrane swellings [67], and in other pathological conditions such as gangliosidoses [68, 69], multiple sclerosis [70], and a number of other neurological conditions [47].

How myelin accommodates structural changes (remodeling) during myelination and demyelination is largely not understood. During myelination, myelin membranes need to bend to render the spiraled, and compacted structure of the mature sheath. Along these lines, during demyelination, myelin lamellae likely undergo analogous "pathogenic" remodeling that weakens and disrupts the highly-ordered myelin sheaths, leading to areas of disruption and myelin loss. In this respect, our results provide a foundational framework to begin studying myelin vulnerability to other myelin components including inverted coned polar GSLs, cholesterol, and myelin proteins. Our study predicts that changes in the local concentration of these molecules may trigger effects not only altering lipid raft behavior [6], but also the fluidity and stability of the myelin sheath.

## Significance statement

The direct participation of neurotoxic sphingolipids such as psychosine in the destruction of myelin in genetic leukodystrophies such as Krabbe's disease remains unaddressed. Here we show that psychosine accumulates in myelin membranes from the animal model of Krabbe's disease causing localized areas of higher rigidity in the membrane. Furthermore, psychosine facilitates the loss of myelin by microvesiculation. This study reveals that abnormal accumulation of a myelin sphingolipid can cause direct structural changes leading to destabilization and destruction of myelin. The implications of local increments of myelin components in vulnerability in other myelin diseases is discussed.

## Supporting information

S1 Fig FRAP analysis of TopFluor-cholesterol mobility in red blood cells. **A)** Delayed time to recover after photobleaching by TopFluor-cholesterol (TpF-chol). **B)** Mobile fraction of TpF-chol is reduced in RBCs treated with 2 μM psychosine. **C)** Half-life measurements are increased in WT RBCs pre-incubated with psychosine compared to vehicle controls. Results are the result of 2–3 independent experiments and are reported as the mean ± SEM. *, *p* <0.05.
(TIF)

S1 Movie Psychosine induces vesiculation. Red blood cells were labeled with BODIPY-SM as in Fig 5 and were exposed to 2 μM psychosine on stage. Images were acquired every 5 min for 60 min by live confocal microscopy. Vesicles progressively appeared in psychosine treated cells in a greater extent than in vehicle-treated cells.
(MP4)

S2 Movie Control of vesiculation in vehicle-treated cells. Red blood cells were labeled with BODIPY-SM as in Fig 5 and were exposed to vehicle (DMSO) on stage. Images were acquired every 5 min for 60 min by live confocal microscopy.
(MP4)

## References

[1] Igisu H, Suzuki K. Progressive accumulation of toxic metabolite in a genetic leukodystrophy. Science. 1984;224(4650):753–5. Epub 1984/05/18. 6719111 10.1126/science.6719111

[2] Bongarzone ER, Escolar ML, Gray SJ, Kafri T, Vite CH, Sands MS. Insights into the Pathogenesis and Treatment of Krabbe Disease. Pediatr Endocrinol Rev. 2016;13 Suppl 1:689–96. 27491217

[3] White AB, Givogri MI, Lopez-Rosas A, Cao H, van Breemen R, Thinakaran G, et al Psychosine accumulates in membrane microdomains in the brain of krabbe patients, disrupting the raft architecture. The Journal of neuroscience: the official journal of the Society for Neuroscience. 2009;29(19):6068–77. 19439584 10.1523/JNEUROSCI.5597-08.2009 PMC6665501

[4] Boggs JM, Menikh A, Rangaraj G. Trans interactions between galactosylceramide and cerebroside sulfate across apposed bilayers. Biophys J. 2000;78(2):874–85. PubMed Central PMCID: PMCPMC1300690. doi: 10.1016/S0006-3495(00)76645-8 10653800 10.1016/S0006-3495(00)76645-8 PMC1300690

[5] Westerlund B, Slotte JP. How the molecular features of glycosphingolipids affect domain formation in fluid membranes. Biochim Biophys Acta. 2009;1788(1):194–201. Epub 2008/12/17. doi: 10.1016/j.bbamem.2008.11.010 19073136 10.1016/j.bbamem.2008.11.010

[6] Lingwood D, Simons K. Lipid rafts as a membrane-organizing principle. Science. 2010;327(5961):46–50. doi: 10.1126/science.1174621 20044567 10.1126/science.1174621

[7] Mukherjee S, Maxfield FR. Lipid and cholesterol trafficking in NPC. Biochimica et biophysica acta. 2004;1685(1–3):28–37. doi: 10.1016/j.bbalip.2004.08.009 15465424 10.1016/j.bbalip.2004.08.009

[8] Prinetti A, Loberto N, Chigorno V, Sonnino S. Glycosphingolipid behaviour in complex membranes. Biochimica et biophysica acta. 2009;1788(1):184–93. doi: 10.1016/j.bbamem.2008.09.001 18835549 10.1016/j.bbamem.2008.09.001

[9] Jarsch IK, Daste F, Gallop JL. Membrane curvature in cell biology: An integration of molecular mechanisms. The Journal of cell biology. 2016;214(4):375–87. Epub 2016/08/17. PubMed Central PMCID: PMCPMC4987295. doi: 10.1083/jcb.201604003 27528656 10.1083/jcb.201604003 PMC4987295

[10] Carquin M, D'Auria L, Pollet H, Bongarzone ER, Tyteca D. Recent progress on lipid lateral heterogeneity in plasma membranes: From rafts to submicrometric domains. Prog Lipid Res. 2015;62:1–24. doi: 10.1016/j.plipres.2015.12.004 26738447 10.1016/j.plipres.2015.12.004 PMC4851880

[11] O'Meara RW, Ryan SD, Colognato H, Kothary R. Derivation of enriched oligodendrocyte cultures and oligodendrocyte/neuron myelinating co-cultures from post-natal murine tissues. J Vis Exp. 2011;(54). PubMed Central PMCID: PMCPMC3217647. 10.3791/3324 PMC3217647 21876528

[12] Pituch KC, Moyano AL, Lopez-Rosas A, Marottoli FM, Li G, Hu C, et al Dysfunction of platelet-derived growth factor receptor alpha (PDGFRalpha) represses the production of oligodendrocytes from arylsulfatase A-deficient multipotential neural precursor cells. J Biol Chem. 2015;290(11):7040–53. PubMed Central PMCID: PMCPMC4358127. doi: 10.1074/jbc.M115.636498 25605750 10.1074/jbc.M115.636498 PMC4358127

[13] Tyteca D, D'Auria L, Van Der Smissen P, Medts T, Carpentier S, Monbaliu JC, et al Three unrelated sphingomyelin analogs spontaneously cluster into plasma membrane micrometric domains. Biochimica et biophysica acta. 2010;1798(5):909–27. doi: 10.1016/j.bbamem.2010.01.021 20123084 10.1016/j.bbamem.2010.01.021

[14] Leppimaki P, Mattinen J, Slotte JP. Sterol-induced upregulation of phosphatidylcholine synthesis in cultured fibroblasts is affected by the double-bond position in the sterol tetracyclic ring structure. European journal of biochemistry. 2000;267(21):6385–94. 11029581 10.1046/j.1432-1327.2000.01726.x

[15] Galbiati F, Basso V, Cantuti L, Givogri MI, Lopez-Rosas A, Perez N, et al Autonomic denervation of lymphoid organs leads to epigenetic immune atrophy in a mouse model of Krabbe disease. The Journal of neuroscience: the official journal of the Society for Neuroscience. 2007;27(50):13730–8. 18077684 10.1523/JNEUROSCI.3379-07.2007 PMC6673629

[16] Parnham MJ, Wetzig H. Toxicity screening of liposomes. Chem Phys Lipids. 1993;64(1–3):263–74. 8242838 10.1016/0009-3084(93)90070-j

[17] D'Auria L, Fenaux M, Aleksandrowicz P, Van Der Smissen P, Chantrain C, Vermylen C, et al Micrometric segregation of fluorescent membrane lipids: relevance for endogenous lipids and biogenesis in erythrocytes. Journal of lipid research. 2013;54(4):1066–76. PubMed Central PMCID: PMC3605983. doi: 10.1194/jlr.M034314 23322884 10.1194/jlr.M034314 PMC3605983

[18] Norton WT, Poduslo SE. Myelination in rat brain: method of myelin isolation. Journal of neurochemistry. 1973;21(4):749–57. 4271082 10.1111/j.1471-4159.1973.tb07519.x

[19] Kostova EB, Beuger BM, Klei TR, Halonen P, Lieftink C, Beijersbergen R, et al Identification of signalling cascades involved in red blood cell shrinkage and vesiculation. Biosci Rep. 2015;35(2). PubMed Central PMCID: PMCPMC4400636. 10.1042/BSR20150019 PMC4400636 25757360

[20] Saldanha C, Santos NC, Martins-Silva J. Fluorescent probes DPH, TMA-DPH and C17-HC induce erythrocyte exovesiculation. J Membr Biol. 2002;190(1):75–82. doi: 10.1007/s00232-002-1025-5 12422273 10.1007/s00232-002-1025-5

[21] Kuhry JG, Duportail G, Bronner C, Laustriat G. Plasma membrane fluidity measurements on whole living cells by fluorescence anisotropy of trimethylammoniumdiphenylhexatriene. Biochimica et biophysica acta. 1985;845(1):60–7. 3978130 10.1016/0167-4889(85)90055-2

[22] Owen DM, Rentero C, Magenau A, Abu-Siniyeh A, Gaus K. Quantitative imaging of membrane lipid order in cells and organisms. Nature protocols. 2012;7(1):24–35. 10.1038/nprot.2011.419 22157973

[23] Cheng ZJ, Singh RD, Sharma DK, Holicky EL, Hanada K, Marks DL, et al Distinct mechanisms of clathrin-independent endocytosis have unique sphingolipid requirements. Mol Biol Cell. 2006;17(7):3197–210. doi: 10.1091/mbc.E05-12-1101 16672382 10.1091/mbc.E05-12-1101 PMC1552047

[24] Marsh JB, Weinstein DB. Simple charring method for determination of lipids. Journal of lipid research. 1966;7(4):574–6. 5965305

[25] Givogri MI, Costa RM, Schonmann V, Silva AJ, Campagnoni AT, Bongarzone ER. Central nervous system myelination in mice with deficient expression of Notch1 receptor. Journal of neuroscience research. 2002;67(3):309–20. Epub 2002/01/29. doi: 10.1002/jnr.10128 11813235 10.1002/jnr.10128

[26] White AB, Galbiati F, Givogri MI, Lopez Rosas A, Qiu X, van Breemen R, et al Persistence of psychosine in brain lipid rafts is a limiting factor in the therapeutic recovery of a mouse model for Krabbe disease. Journal of neuroscience research. 2011;89(3):352–64. PubMed Central PMCID: PMC3064524. doi: 10.1002/jnr.22564 21259322 10.1002/jnr.22564 PMC3064524

[27] Kobayashi T, Yamanaka T, Jacobs JM, Teixeira F, Suzuki K. The Twitcher mouse: an enzymatically authentic model of human globoid cell leukodystrophy (Krabbe disease). Brain research. 1980;202(2):479–83. Epub 1980/12/08. 7437911 10.1016/0006-8993(80)90159-6

[28] Duchen LW, Eicher EM, Jacobs JM, Scaravilli F, Teixeira F. Hereditary leucodystrophy in the mouse: the new mutant twitcher. Brain: a journal of neurology. 1980;103(3):695–710. Epub 1980/09/01. 7417782 10.1093/brain/103.3.695

[29] Gregoire A, Perier O, Dustin P Jr. Metachromatic leukodystrophy, an electron microscopic study. Journal of neuropathology and experimental neurology. 1966;25(4):617–36. Epub 1966/10/01. 5922555 10.1097/00005072-196610000-00008

[30] Cravioto H. In vivo and in vitro studies of metachromatic leukodystrophy. Journal of neuropathology and experimental neurology. 1967;26(1):157–8. Epub 1967/01/01. 6022146

[31] Meier C, Bischoff A. Sequence of morphological alterations in the nervous system of metachromatic leucodystrophy. Light- and electronmicroscopic observations in the central and peripheral nervous system in a prenatally diagnosed foetus of 22 weeks. Acta neuropathologica. 1976;36(4):369–79. Epub 1976/12/21. 1015243 10.1007/BF00699642

[32] Takahashi H, Suzuki K. Demyelination in the spinal cord of murine globoid cell leukodystrophy (the twitcher mouse). Acta neuropathologica. 1984;62(4):298–308. Epub 1984/01/01. 6730907 10.1007/BF00687612

[33] Fontaine G, Resibois A, Tondeur M, Jonniaux G, Farriaux JP, Voet W, et al Gangliosidosis with total hexosaminidase deficiency: clinical, biochemical and ultrastructural studies and comparison with conventional cases of Tay-Sachs disease. Acta neuropathologica. 1973;23(2):118–32. Epub 1973/01/30. 4349527 10.1007/BF00685766

[34] Pellissier JF, Berard-Badier M, Pinsard N. Farber's disease in two siblings, sural nerve and subcutaneous biopsies by light and electron microscopy. Acta neuropathologica. 1986;72(2):178–88. Epub 1986/01/01. 3103372 10.1007/BF00685981

[35] Prineas JW, Kwon EE, Cho ES, Sharer LR. Continual breakdown and regeneration of myelin in progressive multiple sclerosis plaques. Annals of the New York Academy of Sciences. 1984;436:11–32. Epub 1984/01/01. 6598010 10.1111/j.1749-6632.1984.tb14773.x

[36] Zhu H, Lopez-Rosas A, Qiu X, Van Breemen RB, Bongarzone ER. Detection of the neurotoxin psychosine in samples of peripheral blood: application in diagnostics and follow-up of Krabbe disease. Archives of pathology & laboratory medicine. 2012;136(7):709–10. PubMed Central PMCID: PMC3664193. 22742542 10.5858/arpa.2011-0667-LE PMC3664193

[37] Turgeon CT, Orsini JJ, Sanders KA, Magera MJ, Langan TJ, Escolar ML, et al Measurement of psychosine in dried blood spots—a possible improvement to newborn screening programs for Krabbe disease. Journal of inherited metabolic disease. 2015;38(5):923–9. doi: 10.1007/s10545-015-9822-z 25762404 10.1007/s10545-015-9822-z

[38] D'Auria L, Van der Smissen P, Bruyneel F, Courtoy PJ, Tyteca D. Segregation of fluorescent membrane lipids into distinct micrometric domains: evidence for phase compartmentation of natural lipids? PloS one. 2011;6(2):e17021 PubMed Central PMCID: PMC3046177. doi: 10.1371/journal.pone.0017021 21386970 10.1371/journal.pone.0017021 PMC3046177

[39] Kenworthy AK, Nichols BJ, Remmert CL, Hendrix GM, Kumar M, Zimmerberg J, et al Dynamics of putative raft-associated proteins at the cell surface. The Journal of cell biology. 2004;165(5):735–46. PubMed Central PMCID: PMC2172371. doi: 10.1083/jcb.200312170 15173190 10.1083/jcb.200312170 PMC2172371

[40] Tyteca D, D'Auria L, Der Smissen PV, Medts T, Carpentier S, Monbaliu JC, et al Three unrelated sphingomyelin analogs spontaneously cluster into plasma membrane micrometric domains. Biochimica et biophysica acta. 2010;1798(5):909–27. doi: 10.1016/j.bbamem.2010.01.021 20123084 10.1016/j.bbamem.2010.01.021

[41] D'Auria L, Deleu M, Dufour S, Mingeot-Leclercq MP, Tyteca D. Surfactins modulate the lateral organization of fluorescent membrane polar lipids: A new tool to study drug:membrane interaction and assessment of the role of cholesterol and drug acyl chain length. Biochimica et biophysica acta. 2013;1828(9):2064–73. doi: 10.1016/j.bbamem.2013.05.006 23685123 10.1016/j.bbamem.2013.05.006

[42] Yechiel E, Edidin M. Micrometer-scale domains in fibroblast plasma membranes. The Journal of cell biology. 1987;105(2):755–60. PubMed Central PMCID: PMC2114748. 3624308 10.1083/jcb.105.2.755 PMC2114748

[43] D'Auria L, Bongarzone ER. Fluid levity of the cell: Role of membrane lipid architecture in genetic sphingolipidoses. Journal of neuroscience research. 2016;94(11):1019–24. Epub 2016/09/18. PubMed Central PMCID: PMCPMC5027976. doi: 10.1002/jnr.23750 27638586 10.1002/jnr.23750 PMC5027976

[44] Kuhry JG, Fonteneau P, Duportail G, Maechling C, Laustriat G. TMA-DPH: a suitable fluorescence polarization probe for specific plasma membrane fluidity studies in intact living cells. Cell Biophys. 1983;5(2):129–40. doi: 10.1007/BF02796139 6197175 10.1007/BF02796139

[45] Florine-Casteel K, Lemasters JJ, Herman B. Lipid order in hepatocyte plasma membrane blebs during ATP depletion measured by digitized video fluorescence polarization microscopy. FASEB journal: official publication of the Federation of American Societies for Experimental Biology. 1991;5(7):2078–84. Epub 1991/04/01. 2010060 10.1096/fasebj.5.7.2010060

[46] Awojoodu AO, Keegan PM, Lane AR, Zhang Y, Lynch KR, Platt MO, et al Acid sphingomyelinase is activated in sickle cell erythrocytes and contributes to inflammatory microparticle generation in SCD. Blood. 2014;124(12):1941–50. PubMed Central PMCID: PMCPMC4168349. doi: 10.1182/blood-2014-01-543652 25075126 10.1182/blood-2014-01-543652 PMC4168349

[47] Thompson AG, Gray E, Heman-Ackah SM, Mager I, Talbot K, Andaloussi SE, et al Extracellular vesicles in neurodegenerative disease—pathogenesis to biomarkers. Nat Rev Neurol. 2016;12(6):346–57. doi: 10.1038/nrneurol.2016.68 27174238 10.1038/nrneurol.2016.68

[48] Grisendi G, Finetti E, Manganaro D, Cordova N, Montagnani G, Spano C, et al Detection of microparticles from human red blood cells by multiparametric flow cytometry. Blood transfusion = Trasfusione del sangue. 2015;13(2):274–80. PubMed Central PMCID: PMCPMC4385076. doi: 10.2450/2014.0136-14 25369588 10.2450/2014.0136-14 PMC4385076

[49] Nagara H, Kobayashi T, Suzuki K, Suzuki K. The twitcher mouse: normal pattern of early myelination in the spinal cord. Brain research. 1982;244(2):289–94. Epub 1982/07/29. 7116177 10.1016/0006-8993(82)90087-7

[50] Taniike M, Suzuki K. Spacio-temporal progression of demyelination in twitcher mouse: with clinico-pathological correlation. Acta neuropathologica. 1994;88(3):228–36. Epub 1994/01/01. 7528964 10.1007/BF00293398

[51] Louis JC, Magal E, Muir D, Manthorpe M, Varon S. CG-4, a new bipotential glial cell line from rat brain, is capable of differentiating in vitro into either mature oligodendrocytes or type-2 astrocytes. Journal of neuroscience research. 1992;31(1):193–204. doi: 10.1002/jnr.490310125 1613821 10.1002/jnr.490310125

[52] Suzuki K. Twenty five years of the "psychosine hypothesis": a personal perspective of its history and present status. Neurochem Res. 1998;23(3):251–9. 9482237 10.1023/a:1022436928925

[53] Hawkins-Salsbury JA, Parameswar AR, Jiang X, Schlesinger PH, Bongarzone E, Ory DS, et al Psychosine, the cytotoxic sphingolipid that accumulates in globoid cell leukodystrophy, alters membrane architecture. Journal of lipid research. 2013;54(12):3303–11. Epub 2013/09/06. PubMed Central PMCID: PMCPMC3826678. doi: 10.1194/jlr.M039610 24006512 10.1194/jlr.M039610 PMC3826678

[54] Simons K, Ikonen E. Functional rafts in cell membranes. Nature. 1997;387(6633):569–72. doi: 10.1038/42408 9177342 10.1038/42408

[55] Golfetto O, Hinde E, Gratton E. Laurdan fluorescence lifetime discriminates cholesterol content from changes in fluidity in living cell membranes. Biophys J. 2013;104(6):1238–47. PubMed Central PMCID: PMC3602759. doi: 10.1016/j.bpj.2012.12.057 23528083 10.1016/j.bpj.2012.12.057 PMC3602759

[56] Galvan C, Camoletto PG, Cristofani F, Van Veldhoven PP, Ledesma MD. Anomalous surface distribution of glycosyl phosphatidyl inositol-anchored proteins in neurons lacking acid sphingomyelinase. Mol Biol Cell. 2008;19(2):509–22. PubMed Central PMCID: PMCPMC2230584. doi: 10.1091/mbc.E07-05-0439 18032586 10.1091/mbc.E07-05-0439 PMC2230584

[57] von Einem B, Weber P, Wagner M, Malnar M, Kosicek M, Hecimovic S, et al Cholesterol-dependent energy transfer between fluorescent proteins-insights into protein proximity of APP and BACE1 in different membranes in Niemann-Pick type C disease cells. International journal of molecular sciences. 2012;13(12):15801–12. PubMed Central PMCID: PMCPMC3546662. doi: 10.3390/ijms131215801 23443094 10.3390/ijms131215801 PMC3546662

[58] Koike T, Ishida G, Taniguchi M, Higaki K, Ayaki Y, Saito M, et al Decreased membrane fluidity and unsaturated fatty acids in Niemann-Pick disease type C fibroblasts. Biochimica et biophysica acta. 1998;1406(3):327–35. 9630707 10.1016/s0925-4439(98)00019-2

[59] Nishio M, Fukumoto S, Furukawa K, Ichimura A, Miyazaki H, Kusunoki S, et al Overexpressed GM1 suppresses nerve growth factor (NGF) signals by modulating the intracellular localization of NGF receptors and membrane fluidity in PC12 cells. J Biol Chem. 2004;279(32):33368–78. doi: 10.1074/jbc.M403816200 15145933 10.1074/jbc.M403816200

[60] Allt G, Ghabriel MN, Sikri K. Lysophosphatidyl choline-induced demyelination. A freeze-fracture study. Acta neuropathologica. 1988;75(5):456–64. 3376751 10.1007/BF00687132

[61] de Vito G, Cappello V, Tonazzini I, Cecchini M, Piazza V. RP-CARS reveals molecular spatial order anomalies in myelin of an animal model of Krabbe disease. Journal of biophotonics. 2016. Epub 2016/03/19. 10.1002/jbio.201500305 26990139

[62] Cooper RA. Abnormalities of cell-membrane fluidity in the pathogenesis of disease. N Engl J Med. 1977;297(7):371–7. doi: 10.1056/NEJM197708182970707 327326 10.1056/NEJM197708182970707

[63] Shaharabani R, Ram-On M, Avinery R, Aharoni R, Arnon R, Talmon Y, et al Structural Transition in Myelin Membrane as Initiator of Multiple Sclerosis. Journal of the American Chemical Society. 2016;138(37):12159–65. doi: 10.1021/jacs.6b04826 27548321 10.1021/jacs.6b04826

[64] Chauhan NB. Membrane dynamics, cholesterol homeostasis, and Alzheimer's disease. Journal of lipid research. 2003;44(11):2019–29. doi: 10.1194/jlr.R300010-JLR200 12951356 10.1194/jlr.R300010-JLR200

[65] Yang X, Sun GY, Eckert GP, Lee JC. Cellular membrane fluidity in amyloid precursor protein processing. Mol Neurobiol. 2014;50(1):119–29. doi: 10.1007/s12035-014-8652-6 24553856 10.1007/s12035-014-8652-6 PMC6599628

[66] McMahon HT, Boucrot E. Membrane curvature at a glance. Journal of cell science. 2015;128(6):1065–70. Epub 2015/03/17. PubMed Central PMCID: PMCPmc4359918. doi: 10.1242/jcs.114454 25774051 10.1242/jcs.114454 PMC4359918

[67] Castelvetri LC, Givogri MI, Zhu H, Smith B, Lopez-Rosas A, Qiu X, et al Axonopathy is a compounding factor in the pathogenesis of Krabbe disease. Acta neuropathologica. 2011;122(1):35–48. PubMed Central PMCID: PMC3690521. doi: 10.1007/s00401-011-0814-2 21373782 10.1007/s00401-011-0814-2 PMC3690521

[68] Purpura DP. Ectopic dendritic growth in mature pyramidal neurones in human ganglioside storage disease. Nature. 1978;276(5687):520–1. 102998 10.1038/276520a0

[69] Purpura KP, Baker HJ. Neurite induction in mature cortical neurones in feline GM1-ganglioside storage disease. Nature. 1977;266(5602):553–4. 404568 10.1038/266553a0

[70] Dutta R, Trapp BD. Mechanisms of neuronal dysfunction and degeneration in multiple sclerosis. Prog Neurobiol. 2011;93(1):1–12. PubMed Central PMCID: PMCPMC3030928. doi: 10.1016/j.pneurobio.2010.09.005 20946934 10.1016/j.pneurobio.2010.09.005 PMC3030928

