# Supplementary figures and images for: Psychosine enhances the shedding of membrane microvesicles: Implications in demyelination in Krabbe’s disease
Source: PLoS One. 2017 May 22;12(5):e0178103. doi: 10.1371/journal.pone.0178103 (PMC5439731; doi:10.1371/journal.pone.0178103)

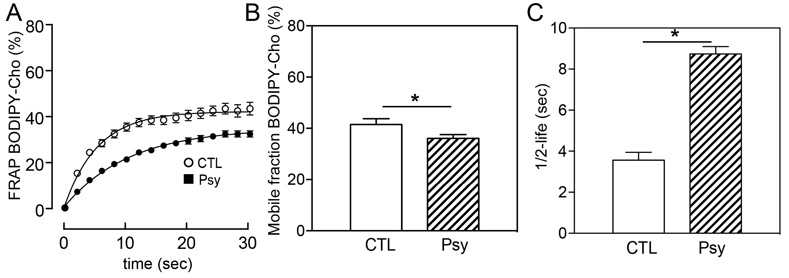

Supplement: S1 Fig — A) Delayed time to recover after photobleaching by TopFluor-cholesterol (TpF-chol). B) Mobile fraction of TpF-chol is reduced in RBCs treated with 2 μM psychosine. C) Half-life measurements are increased in WT RBCs pre-incubated with psychosine compared to vehicle controls. Results are the result of 2–3 independent experiments and are reported as the mean ± SEM. *, p <0.05. (TIF) [file pone.0178103.s001.tif]
